# Supplementary material for: Fluoroquinolone resistance-conferring gyrA variants alter the fitness cost and potentiate the resistance of the zoliflodacin resistance mutation gyrBD429N in Neisseria gonorrhoeae
Source: Antimicrob Agents Chemother. 2026 Aug 19;70(10):e00766-26. doi: 10.1128/aac.00766-26 (PMC13688830; doi:10.1128/aac.00766-26)
Supplement: Supplementary file 1 [file aac.00766-26-s0001.docx]

**Fluoroquinolone resistance-conferring *gyrA* variants alter the fitness cost and potentiate the resistance of the zoliflodacin resistance mutation *gyrB*^D429N^ in *Neisseria gonorrhoeae***

Aditi Mukherjee^1^, Sofia OP Blomqvist^1^, David Helekal^1^, Daniel HF Rubin^1^, Bailey Bowcutt^1^, Samantha G Palace^1^, Yonatan H Grad^1,2^

1. Department of Immunology and Infectious Diseases, Harvard T. H. Chan School of Public Health, Boston, Massachusetts, USA
2. Corresponding author

**Corresponding author’s contact information:** Yonatan Grad, Department of Immunology and Infectious Diseases, Harvard T.H. Chan School of Public Health, Boston, MA 02115, USA; [ygrad@hsph.harvard.edu](mailto:ygrad@hsph.harvard.edu)

**Supplementary Table 1:** Plasmids, *N. gonorrhoeae* strains and primers used in this study.

| **Plasmids** | **Description** | **Reference** |
| --- | --- | --- |
| pAM_3 | pUC19 with CM^R^ cassette + homology to clone *gyrA*^91S/95D^ | ^1^ |
| pDR53 | A derivative of pDR1^1^ without Lac promoter and the regulatory elements + added multi cloning site (MCS) | ^2^ |
| pBC1 | A derivative of pDR53 that inserts a random 20-mer barcode into the neutral site between *aspC* and *lctP* | This study |

| **Strains** | **Genotype** | **Reference** |
| --- | --- | --- |
| GCGS0481_*gyrA*^91F/95G^ (Parent) | *gyrA*^91F/95G^, *parC*^S87R^ | ^3^ |
| GCGS0481_*gyrA*^91S/95D^ | CM^R^, *gyrA*^91S/95D^, *parC*^S87R^ | ^1^ |
| GCGS0481_*gyrA*^91S/95G^ | CM^R^, *gyrA*^91S/95G^, *parC*^S87R^ | This study |
| GCGS0481_*gyrA*^91S/95A^ | CM^R^, *gyrA*^91S/95A^, *parC*^S87R^ | This study |
| GCGS0481_*gyrA*^91S/95N^ | CM^R^, *gyrA*^91S/95N^, *parC*^S87R^ | This study |
| GCGS0481_*gyrA*^91F/95D^ | CM^R^, *gyrA*^91F/95D^, *parC*^S87R^ | This study |
| GCGS0481_*gyrA*^91F/95G^ | CM^R^, *gyrA*^91F/95G^, *parC*^S87R^ | This study |
| GCGS0481_*gyrA*^91F/95A^ | CM^R^, *gyrA*^91F/95A^, *parC*^S87R^ | This study |
| GCGS0481_*gyrA*^91F/95N^ | CM^R^, *gyrA*^91F/95N^, *parC*^S87R^ | This study |
| GCGS0481_*gyrA*^91S/95D^_*gyrB*^D429N^ | CM^R^, *gyrA*^91S/95D^, *parC*^S87R^, *gyrB*^D429N^ | This study |
| GCGS0481_*gyrA*^91S/95G^_*gyrB*^D429N^ | CM^R^, *gyrA*^91S/95G^, *parC*^S87R^, *gyrB*^D429N^ | This study |
| GCGS0481_*gyrA*^91S/95A^_*gyrB*^D429N^ | CM^R^, *gyrA*^91S/95A^, *parC*^S87R^, *gyrB*^D429N^ | This study |
| GCGS0481_*gyrA*^91S/95N^_*gyrB*^D429N^ | CM^R^, *gyrA*^91S/95N^, *parC*^S87R^, *gyrB*^D429N^ | This study |
| GCGS0481_*gyrA*^91F/95D^_*gyrB*^D429N^ | CM^R^, *gyrA*^91F/95D^, *parC*^S87R^, *gyrB*^D429N^ | This study |
| GCGS0481_*gyrA*^91F/95G^_*gyrB*^D429N^ | CM^R^, *gyrA*^91F/95G^, *parC*^S87R^, *gyrB*^D429N^ | This study |
| GCGS0481_*gyrA*^91F/95A^_*gyrB*^D429N^ | CM^R^, *gyrA*^91F/95A^, *parC*^S87R^, *gyrB*^D429N^ | This study |
| GCGS0481_*gyrA*^91F/95N^_*gyrB*^D429N^ | CM^R^, *gyrA*^91F/95N^, *parC*^S87R^, *gyrB*^D429N^ | This study |

| **Primers** | **Sequence (Annealing)** | **Description** |
| --- | --- | --- |
| AM_5 | ATGCCGTCTGAAATCCCGACAAATTCGCTTC | Forward to amplify *gyrA* allele from *Ng* gDNA |
| AM_6 | TTGCCTATGGGTTCGATATG | Reverse to amplify *gyrA* allele from *Ng* gDNA |
| AM_1 | ATGCCGTCTGAAATGCGCAATGCAATGGAACG | Forward to amplify *gyrB* allele from *Ng* gDNA |
| AM_2 | GATGACGGTTTTCGCCAACA | Reverse to amplify *gyrB* allele from *Ng* gDNA |
| DR412 | AACCATCCGTTCTTAATTAANNNNNNNNNNNNNNNNNNNNTCTAGAACGCGTCGACGCTT | Forward used to introduce a random 20-mer barcode into the MCS of pDR53 |
| DR413 | AAGCGTCGACGCG | Reverse used to introduce a random 20-mer barcode into the MCS of pDR53 |
| P6 | CGCGCGAGCTGTATGA | Reverse to amplify barcode amplicon in aspC/lctP site in *Ng* genome for screening barcoded clones |
| P7 | GCCGCAATCCGCTATAATCC | Forward to amplify/Sanger sequence barcode amplicon in aspC/lctP site in *Ng* genome for screening barcoded clones |
| P41 | ATGACTGCGCTTACCCTTC | Forward to amplify barcode amplicon in aspC/lctP site in *Ng* genome for Nanopore sequencing |
| P42 | GAGGATATCGGGGAAGAACAG | Reverse to amplify barcode amplicon in aspC/lctP site in *Ng* genome for Nanopore sequencing |

**Supplementary Figure 1:** *In vitro* growth kinetics of isogenic strains with different *gyrA* alleles, measured by spectrophotometry. All isogenic strains were cultured separately in liquid GCP media supplemented with Kellogg’s supplement at a starting absorbance reading at 600nm (OD_600_) = 0.1. OD_600_ readings at 0, 2, 4, 6, and 8 hours timepoint are plotted. (A) A_600_ readings at 0, 2, 4, 6, and 8 hours. (B) Dilutions were plated on GCB-K plates at 0, 2, 4, 6, and 8 hours, colonies were counted after overnight growth, and CFUs were calculated. n = 3, representative of three independent experiments. Error bars represent standard deviation of three biological replicates. Statistical significance comparing each strain to GCGS0481 *gyrA*^91S/95D^ was determined by unpaired two-sided Student’s *t*-test (no significant results found).

**Supplementary Figure 2:** *In vitro* growth kinetics of isogenic strains with *gyrB*^D429N^ in combination with different *gyrA* alleles, measured by spectrophotometry. All isogenic strains were cultured separately in liquid GCP media supplemented with Kellogg’s supplement at a starting absorbance reading at 600nm (OD_600_) = 0.1. OD_600_ readings at 0, 2, 4, 6, and 8 hours timepoint are plotted. (A) A_600_ readings at 0, 2, 4, 6, and 8 hours. (B) Dilutions were plated on GCB-K plates at 0, 2, 4, 6, and 8 hours, colonies were counted after overnight growth, and CFUs were calculated. n = 3, representative of three independent experiments. Error bars represent standard deviation of three biological replicates. Statistical significance comparing each strain to GCGS0481 *gyrA*^91S/95D^ *gyrB*^D429N^ was determined by unpaired two-sided Student’s *t-test* and are indicated *p ≤ 0.05, **p ≤ 0.01, and ***p ≤ 0.001.

**Supplementary Figure 3:** Relative fitness of the GCGS0481 *gyrA*^91S/95D^ strain compared to the *gyrB*^D429N^ mutants in the isogenic *gyrA* mutant panel. Y-axes show the competitive index (CI) of the ciprofloxacin-susceptible strain GCGS0481 *gyrA*^91S/95D^ compared to each *gyrB*^D429N^ mutant. The parental strain carried a kanamycin marker and was cocultured with each unmarked *gyrB*^D429N^ mutant strain. (A) *gyrA*^91S/95D^ *gyrB*^429D^ over *gyrA*^91F/95D^ *gyrB*^429N^: p = 0.99, 0.02, 0.04, 0.02, respectively, for 2, 4, 6, and 8 hours. (B) *gyrA*^91S/95D^ *gyrB*^429D^ over *gyrA*^91F/95G^ *gyrB*^429N^: p = 0.31, 0.04, 0.006, 0.0006, respectively, for 2, 4, 6, and 8 hours. (C) *gyrA*^91S/95D^ *gyrB*^429D^ over *gyrA*^91F/95A^ *gyrB*^429N^: p = 0.97, 0.04, 0.01, 0.008, respectively, for 2, 4, 6, and 8 hours. (D) *gyrA*^91S/95D^ *gyrB*^429D^ over *gyrA*^91F/95N^ *gyrB*^429N^: p = 0.09, 0.001, 0.005, 0.001, respectively, for 2, 4, 6, and 8 hours. (E) *gyrA*^91S/95D^ *gyrB*^429D^ over *gyrA*^91S/95D^ *gyrB*^429N^: p = 0.92, 0.21, 0.6, 0.29, respectively, for 2, 4, 6, and 8 hours. (F) *gyrA*^91S/95D^ *gyrB*^429D^ over *gyrA*^91S/95G^ *gyrB*^429N^: p = 0.32, 0.54, 0.39, 0.98, respectively, for 2, 4, 6, and 8 hours. (G) *gyrA*^91S/95D^ *gyrB*^429D^ over *gyrA*^91S/95A^ *gyrB*^429N^: p = 0.51, 0.16, 0.77, 0.64, respectively, for 2, 4, 6, and 8 hours. (H) *gyrA*^91S/95D^ *gyrB*^429D^ over *gyrA*^91S/95N^ *gyrB*^429N^: p = 0.81, 0.83, 0.61, 0.88, respectively, for 2, 4, 6, and 8 hours. n = 3, representative of three independent experiments performed in absence of any antibiotic pressure. Shown: mean with 95% confidence interval, with statistically significant differences in competitive indices compared to time 0 analyzed using an unpaired Student’s *t-test*, indicated *p ≤ 0.05, **p ≤ 0.01, and ***p ≤ 0.001.

**Supplementary Figure 4:** Relative fitness of each *gyrA* strain relative to its own *gyrB*^D429N^ derivative. Y-axes show the competitive index of each (CI) of each parental GCGS0481 relative to *gyrB*^D429N^ mutants. The parental strain carried a kanamycin marker and was cocultured with its unmarked *gyrB*^D429N^ mutant. (A) *gyrA*^91F/95D^ *gyrB*^429D^: p = 0.96, 0.16, 0.02, 0.02, respectively, for 2, 4, 6, and 8 hours. (B) *gyrA*^91F/95G^ *gyrB*^429D^: p = 0.79, 0.05, 0.001, 0.03, respectively, for 2, 4, 6, and 8 hours. (C) *gyrA*^91F/95A^ *gyrB*^429D^: p = 0.96, 0.29, 0.007, 0.007, respectively, for 2, 4, 6, and 8 hours. (D) *gyrA*^91F/95N^ *gyrB*^429D^: p = 0.49, 0.0007, 0.005, 0.004, respectively, for 2, 4, 6, and 8 hours. (E) *gyrA*^91S/95G^ *gyrB*^429D^: p = 0.3, 0.6, 0.82, 0.76, respectively, for 2, 4, 6, and 8 hours. (F) *gyrA*^91S/95A^ *gyrB*^429D^: p = 0.38, 0.71, 0.18, 0.7, respectively, for 2, 4, 6, and 8 hours. (G) *gyrA*^91S/95N^ *gyrB*^429D^: p = 0.22, 0.21, 0.63, 0.53, respectively, for 2, 4, 6, and 8 hours. n = 3, representative of three independent experiments performed in absence of any antibiotic pressure. Shown: mean with 95% confidence interval. Statistically significant differences in competitive indices compared to time 0 were analyzed using an unpaired Student’s *t-test*, indicated *p ≤ 0.05 and **p ≤ 0.01.

**Supplementary Figure 5:** Relative fitness of the each isogenic *gyrA* mutant strain relative to *gyrA*^91S/95D^ in the absence of *gyrB* mutation. Y-axes show the competitive index (CI) (panels A-G) or fitness difference (panel H) of each *gyrA* mutant relative to the ciprofloxacin-susceptible strain *gyrA*^91S/95D^. In pairwise assays (panels A-G), the *gyrA* mutant carried a kanamycin marker and was cocultured with the unmarked *gyrA*^91S/95D^ strain; shown: mean with 95% confidence interval, with statistically significant differences in competitive indices compared to time 0 analyzed using an unpaired Student’s *t-test*, indicated *p ≤ 0.05, **p ≤ 0.01, ***p ≤ 0.001 and ****p ≤ 0.0001. n = 3, representative of three independent experiments performed in absence of any antibiotic pressure. (A) *gyrA*^91F/95D^: p = 0.75, 0.91, 0.09, 0.0006, respectively, for 2, 4, 6, and 8 hours. (B) *gyrA*^91F/95G^: p = 0.27, 0.24, <0.0001, <0.0001, respectively, for 2, 4, 6, and 8 hours. (C) *gyrA*^91F/95A^: p = 0.66, 0.95, <0.0001, 0.007, respectively, for 2, 4, 6, and 8 hours. (D) *gyrA*^91F/95N^: p = 0.45, 0.3, 0.01, 0.002, respectively, for 2, 4, 6, and 8 hours. (E) *gyrA*^91S/95G^: p = 0.2, 0.44, 0.08, 0.51, respectively, for 2, 4, 6, and 8 hours. (F) *gyrA*^91S/95A^: p = 0.3, 0.81, 0.09, 0.47, respectively, for 2, 4, 6, and 8 hours. (G) *gyrA*^91S/95N^: p = 0.32, 0.11, 0.32, 0.33, respectively, for 2, 4, 6, and 8 hours. (H) Estimated difference in exponential-phase growth rate between *gyrA*^91S/95D^ and each mutant *gyrA* strain, calculated from pooled competition experiments via amplicon sequencing of barcodes; Shown: median and 95% credible interval (CrI) estimate for difference in growth rate, estimated from 2 biological replicates of all barcoded strains grown together (4 barcodes per strain per replicate; timepoints at 0, 2, and 4 hours of coculture).

**Supplementary Figure 6:** Relative fitness of the *gyrA*^91S/95D^ strain compared to other isogenic *gyrA* mutants. Y-axes show the competitive index (CI) of *gyrA*^91S/95D^ relative to each *gyrA* mutant. The *gyrA*^91S/95D^ strain carried a kanamycin marker and was cocultured with unmarked *gyrA* mutant strains. (A) *gyrA*^91S/95D^ over *gyrA*^91F/95D^: p = 0.96, 0.76, 0.13, 0.04, respectively, for 2, 4, 6, and 8 hours. (B) *gyrA*^91S/95D^ over *gyrA*^91F/95G^: p = 0.96, 0.97, 0.02, 0.01, respectively, for 2, 4, 6, and 8 hours. (C) *gyrA*^91S/95D^ over *gyrA*^91F/95A^: p = 0.18, 0.27, 0.02, 0.006, respectively, for 2, 4, 6, and 8 hours. (D) *gyrA*^91S/95D^ over *gyrA*^91F/95N^: p = 0.2, 0.7, 0.01, 0.02, respectively, for 2, 4, 6, and 8 hours. (E) *gyrA*^91S/95D^ over *gyrA*^91S/95G^: p = 0.15, 0.053, 0.35, 0.11, respectively, for 2, 4, 6, and 8 hours. (F) *gyrA*^91S/95D^ over *gyrA*^91S/95A^: p = 0.27, 0.69, 0.22, 0.1, respectively, for 2, 4, 6, and 8 hours. (G) *gyrA*^91S/95D^ over *gyrA*^91S/95N^: p = 0.56, 0.47, 0.34, 0.8, respectively, for 2, 4, 6, and 8 hours. n = 3, representative of three independent experiments performed in absence of any antibiotic pressure. Shown: mean with 95% confidence interval. Statistically significant differences in competitive indices compared to time 0 were analyzed using an unpaired Student’s *t-test*, indicated *p ≤ 0.05 and **p ≤ 0.01.

**Supplementary Figure 7:** Relative fitness of the *gyrA*^91F/95D^ genotype compared to isogenic *gyrA*^S91F^ strains with various *gyrA*^95^ mutations, alone (A-C) or in combination with the *gyrB*^D429N^ mutation (D-F). The *gyrA*^S91F^ strain carried a kanamycin marker and was cocultured with the unmarked mutant strains with various *gyrA*^95^ alleles in the presence or absence of *gyrB*^D429N^ mutation. (A) *gyrA*^91F/95D^ over *gyrA*^91F/95G^: p = 0.58, 0.84, 0.31, 0.65 respectively, for 2, 4, 6, and 8 hours. (B) *gyrA*^91F/95D^ over *gyrA*^91F/95A^: p = 0.35, 0.15, 0.88, 0.31, respectively, for 2, 4, 6, and 8 hours. (C) *gyrA*^91F/95D^ over *gyrA*^91F/95N^: p = 0.21, 0.1, 0.1, 0.14, respectively, for 2, 4, 6, and 8 hours. (D) *gyrA*^91F/95D^ *gyrB*^429N^ over *gyrA*^91F/95G^ *gyrB*^429N^: p = 0.07, 0.13, 0.02, 0.0006, respectively, for 2, 4, 6, and 8 hours. (E) *gyrA*^91F/95D^ *gyrB*^429N^ over *gyrA*^91F/95A^ *gyrB*^429N^: p = 0.62, 0.96, 0.09, 0.002, respectively, for 2, 4, 6, and 8 hours. (F) *gyrA*^91F/95D^ *gyrB*^429N^ over *gyrA*^91F/95N^ *gyrB*^429N^: p = 0.7, 0.34, 0.05, 0.04, respectively, for 2, 4, 6, and 8 hours. n = 3, representative of three independent experiments performed in absence of any antibiotic pressure. Shown: mean with 95% confidence interval. Statistically significant differences in competitive indices compared to time 0 were analyzed using an unpaired Student’s *t-test*, indicated *p ≤ 0.05, **p ≤ 0.01, and ***p ≤ 0.001.

**References**

1.	Helekal, D. *et al.* Quantifying the real-world impact of antibiotic use and genetic determinants of resistance on gonococcal dynamics. *Nat Microbiol* (2026).

2.	Rubin, D.H.F. *et al.* CanB is a metabolic mediator of antibiotic resistance in Neisseria gonorrhoeae. *Nat Microbiol* **8**, 28-39 (2023).

3.	Grad, Y.H. *et al.* Genomic Epidemiology of Gonococcal Resistance to Extended-Spectrum Cephalosporins, Macrolides, and Fluoroquinolones in the United States, 2000-2013. *J Infect Dis* **214**, 1579-1587 (2016).
